# Comparison of antimicrobial resistance genes on the ocular surface of patients with corneal infections in California and Malawi

**DOI:** 10.1093/inthealth/ihaf042

**Published:** 2025-04-29

**Authors:** Gerami D Seitzman, Khumbo Kalua, Esther S Misanjo, Cindi Chen, Kevin Ouimette, Lina Zhong, YuHeng Liu, Danny Yu, Thomas Abraham, Nathaniel Wu, Daisy Yan, Thomas M Lietman, Armin Hinterwirth, Thuy Doan

**Affiliations:** Francis I. Proctor Foundation, University of California, San Francisco, CA 94158, USA; Department of Ophthalmology, University of California, San Francisco, CA 94158 USA; Department of Ophthalmology, Kamuzu College of Health Sciences, Blantyre, Malawi; Department of Ophthalmology, Kamuzu College of Health Sciences, Blantyre, Malawi; Francis I. Proctor Foundation, University of California, San Francisco, CA 94158, USA; Francis I. Proctor Foundation, University of California, San Francisco, CA 94158, USA; Francis I. Proctor Foundation, University of California, San Francisco, CA 94158, USA; Francis I. Proctor Foundation, University of California, San Francisco, CA 94158, USA; Francis I. Proctor Foundation, University of California, San Francisco, CA 94158, USA; Francis I. Proctor Foundation, University of California, San Francisco, CA 94158, USA; Francis I. Proctor Foundation, University of California, San Francisco, CA 94158, USA; Francis I. Proctor Foundation, University of California, San Francisco, CA 94158, USA; Francis I. Proctor Foundation, University of California, San Francisco, CA 94158, USA; Department of Ophthalmology, University of California, San Francisco, CA 94158 USA; Francis I. Proctor Foundation, University of California, San Francisco, CA 94158, USA; Francis I. Proctor Foundation, University of California, San Francisco, CA 94158, USA; Department of Ophthalmology, University of California, San Francisco, CA 94158 USA

**Keywords:** aminoglycoside resistance, antimicrobial resistance, infectious keratitis, macrolide resistance, Malawi, RNA-deep sequence analysis

## Abstract

**Background:**

Antimicrobial resistance (AMR) determinants on the ocular surface may contribute to poor treatment responses.

**Methods:**

An exploratory comparative analysis of ocular surface AMR determinants, as determined by RNA-sequencing (RNA-seq), on patients presenting with corneal infections at the Proctor Medical Clinic at the University of California San Francisco in San Francisco, CA, USA, and the Queen Elizabeth Central Hospital at the Department of Ophthalmology of Kamuzu College of Health Sciences in Blantyre, Malawi, was conducted. All patients underwent swabbing of three sites on the ocular surface: cornea, ipsilateral conjunctiva, and contralateral conjunctiva.

**Results:**

Mutations conferring macrolide resistance were present on the ocular surface in 58% (95% CI 44 to 71%) of the participants in Malawi and 32% (95% CI 20 to 46%) of the participants in San Francisco. Aminoglycosides resistance genes were also common on the ocular surface with 58% (95% CI 44 to 71%) prevalence in Malawi and 21% (95% CI 12 to 35%) in San Francisco. AMR was associated with poorer visual outcomes in a subset of patients.

**Conclusions:**

As determined by RNA-seq, ocular surface AMR gene mutations are common in patients with infectious keratitis. Surveillance may be important for infectious keratitis treatment selection as well as providing guidance for antibiotic stewardship.

## Introduction

Ocular surface infections are a leading cause of vision morbidity worldwide with pathogens varying by location.^1^ The ideal treatment strategy includes rapid identification of causative organisms followed by appropriate topical antimicrobial administration.^2^ However, in clinical practice, microbiologic determination of etiology is rarely investigated, and broad-spectrum antibiotics are empirically initiated. Both topical and systemic antibiotic prescription patterns vary worldwide. In the USA, antibiotics require a doctor's prescription. Despite this requirement, the continued consensus is that antibiotics are consistently overused.^3^ In other parts of the world, such as Malawi and surrounding countries in Africa, antibiotic dispensing without prescription is common.^4,5^ This may further contribute to an antibiotic overuse environment. Antibiotic use is known to select for antibiotic resistance.^6,7^ It is unknown how different antibiotic practice patterns affect the carriage of antimicrobial resistance (AMR) determinants on the ocular surface.

AMR can be assessed phenotypically or genotypically. Phenotypic resistance measures how well organisms survive in the presence of antimicrobial medication. Genotypic resistance indicates that gene mutations are present that carry the potential to affect the survival of the organism in the presence of antibiotics. There is a significant correlation between these two types of resistance.^8,9^ The precise relationship between ocular surface genotypic and phenotypic resistance is unknown and likely varies with organism and medication. It is also not well-defined if and how the presence of AMR on the ocular surface affects treatment outcomes in the setting of corneal infections. In bacterial keratitis, phenotypic resistance to topical antibiotics is associated with a worse clinical outcome.^10^ In general, it is assumed that in the setting of infectious keratitis, treatment with antimicrobials is most effective if AMR determinants are absent from the ocular surface.

capriCORN (Comprehensive Analysis of Pathogens, Resistomes, and Inflammatory-markers in the CORNea) is an ongoing international study where patients with infectious keratitis are swabbed for unbiased RNA-sequencing (RNA-seq). In this study, swabs from the ocular surface, which includes the infected cornea as well as bilateral conjunctivae, were obtained and assessed for both pathogen identification as well as the presence of AMR genes. The Proctor Foundation at the University of California San Francisco (UCSF), USA, and the Department of Ophthalmology of Kamuzu College of Health Sciences in Blantyre, Malawi, Africa, are participating capriCORN sites.

## Methods

Patients presenting to the outpatient ophthalmology services with a clinical diagnosis of infectious keratitis at UCSF and the Queen Elizabeth Central Hospital, Department of Ophthalmology of Kamuzu College of Health Sciences, were examined, consented for capriCORN and underwent sample collection during the same visit. All patients had swabs obtained from their affected cornea, ipsilateral conjunctiva and contralateral conjunctiva for RNA-seq. In this study, the term ‘ocular surface’ is used to indicate that AMR determinants were detected on at least one of these three swabs.

Sterile polyester applicators (Puritan, Guilford, ME, USA) were used to swab the cornea and conjunctivae. The swabs were stored in DNA/RNA-Shield (Zymo Research, Irvine, CA, USA) and transferred to a -80°C freezer. Samples from the Kamuzu College of Health Sciences were shipped to the Proctor Foundation for sample processing. Samples were de-identified and randomized prior to sample processing. All laboratory personnel handling samples and interpreting data were masked to all identifying and all clinical data. For this exploratory analysis, the sample size was based on the available cohort from each site at the time of analysis and was not prespecified.

RNA-seq library preparation, sequencing and bioinformatics analyses for AMR have been previously described.^11,12^ Briefly, 5 µl of extracted RNA of each sample was converted to cDNA and sequencing libraries were prepared using the NEBNext ULTRA II RNA Library Prep Kit for Illumina (New England Biolabs) and sequenced on the NovaSeq X system (Illumina, San Diego, CA, USA) using 150-nucleotide paired-end sequencing. Host reads were removed and the remaining non-host reads were filtered for quality. Those non-host reads passing the quality filter were aligned to the MEGARes reference antimicrobial database (version 3.0), using the Burrows-Wheeler Aligner with default settings. The MEGARes database includes >8000 curated AMR gene accessions from sources that include the Comprehensive Antibiotic Resistance Database (or CARD), the National Center for Biotechnology Information (NCBI)’s Bacterial Antimicrobial Resistance Reference Gene Database and ResFinder.^13^ An AMR genetic determinant is called if it meets >80% nucleotide identity between the query sequence and the reference AMR gene. Any hits that fail Single nucleotide polymorphism (SNP)-confirmation (for genes that require it) are excluded. To correct for sequencing depth, the number of matched AMR determinants were normalized to the total number of non-host reads within each sample prior to comparing relative abundance between samples. Visual acuity measurements were obtained with the Snellen eye chart, using pinhole testing to approximate best-corrected acuity. Snellen acuity was then converted to the logarithm of the minimum angle of resolution (LogMAR) scale for statistical analysis.

For both the UCSF and Malawi cohorts, multivariable linear regression was performed to assess the relationship between the number of AMR determinants present on the ocular surface and visual acuity at 4 wk, controlling for visual acuity at presentation. An additional multivariable linear regression model was fit to examine this relationship while adjusting for both visual acuity at presentation and pathogen presence (acanthamoeba, bacteria, fungus or none). Here, ‘no pathogen presence’ was set as the reference variable. Statistical analyses were performed using R version 4.3.1 (R Foundation for Statistical Computing, Vienna, Austria). For this exploratory analysis, multivariable linear regression models were fitted using the *lm ()* function from the *stats* package.^14^ p<0.05 (two-tailed) values were considered notable and were not corrected for multiple comparisons.

## Results

Forty-seven patients from UCSF and 50 patients from the Department of Ophthalmology of Kamuzu College of Health Sciences were included in this analysis (Table 1). Of the UCSF patients, 96% (95% CI 85 to 100%) presented taking an eyedrop. Of these medications, 84% (95% CI 71 to 93%) were antibacterial, 16% were antifungal (95% CI 6 to 27%), 3% (95% CI 0 to 13%) were antiviral and 29% (95% CI 18 to 43%) were topical steroids. At the Kamuzu College of Health Sciences, 62% (95% CI 48 to 74%) of patients presented for care on an eyedrop. Here, 74% (95% CI 57 to 87%) were antibacterial, 6% were antifungal (95% CI 0 to 22%) and 32% (95% CI 18 to 50%) were topical steroids; no patients were on antivirals and 13% (95% CI 5 to 30%) of the eyedrops were of an unknown category, with the most common reason being that the patient did not recall the name of the eyedrops they were using.

**Table 1.** Demographics and AMR prevalence at San Francisco in the USA and Blantyre in Malawi

	California, USA	Blantyre, Malawi
Total number	47	50
Average age (y±SD)	55±21	40±17
% female	60%	39%
% taking antibacterial medication (95% CI)	81% (67 to 90%)	46% (33 to 60%)
% macrolide AMR gene (95% CI)	32% (20 to 46%)	58% (44 to 71%)
% aminoglycoside AMR gene (95% CI)	21% (12 to 35%)	58% (44 to 71%)
% betalactam AMR gene (95% CI)	0% (0 to 7%)	2% (0 to 11%)
% fluoroquinolone AMR gene (95% CI)	0% (0 to 7%)	2% (0 to 11%)
% cAMP AMR gene (95% CI)	0% (0 to 7%)	0% (0 to 6%)
% rifampin AMR gene (95% CI)	0% (0 to 7%)	0% (0 to 6%)
% sulfonamide AMR gene (95% CI)	0% (0 to 7%)	2% (0 to 11%)
% tetracycline AMR gene (95% CI)	2% (0 to 12%)	2% (0 to 11%)

AMR: antimicrobial resistance; cAMP: cationic antimicrobial peptides.

95% CI, adjusted Wald method.

**Figure 1.**
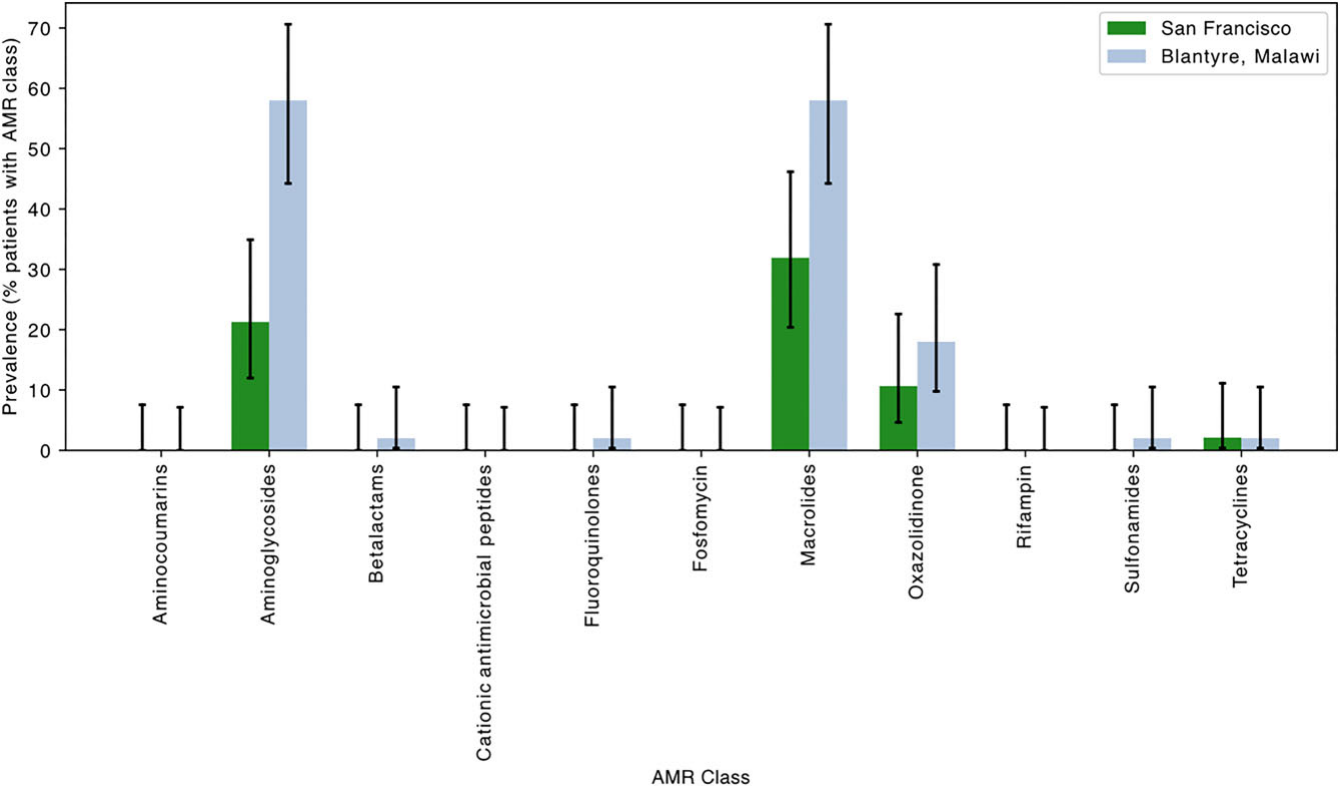
Prevalence of antimicrobial resistance (AMR) genes on the ocular surface of patients in San Francisco, California, USA, and Blantyre, Malawi. Error bars represent 95% confidence interval (CI), Wilson method.

The following results indicate the presence of mutations in genes associated with AMR carried by sampled organisms on the ocular surface, meaning that AMR determinants were detected on at least one of the three ocular surface swabs. These include AMR determinants contained within the host conjunctival microbiome as well as corneal pathogens. On the ocular surface of the UCSF population, 32% (95% CI 20 to 46%) carried macrolide resistance determinants, 21% (95% CI 12 to 35%) carried aminoglycoside resistance determinants and 2% (95% CI 0 to 12%) carried tetracycline resistance determinants (Figure 1). Resistance determinants for beta-lactam, fluoroquinolones, cationic antimicrobial peptides (cAMP), rifampin and sulfonamides were not detected on the ocular surface. On the ocular surface of patients in Malawi, 58% (95% CI 44 to 71%) carried resistance determinants for macrolides and aminoglycosides, although not in the same patients. In addition, 2% (95% CI 0 to 11%) of this population had antibiotic resistance determinants for beta-lactams, fluoroquinolones, sulfonamides and tetracyclines. No AMR determinants were noted for cAMP or rifampin.

At both institutions, the current use of a particular antibiotic class at the time of swabbing did not correlate with the presence of AMR determinants for that antibiotic. At UCSF, of the 15 participants with macrolide resistance determinants on the ocular surface, only one presented taking a topical macrolide, and of the 32 without macrolide resistance determinants, three presented on topical macrolides (p=0.76). Similarly, five of 10 patients carrying aminoglycoside resistance determinants presented on topical aminoglycosides, as did 14 of the 23 patients who did not carry aminoglycoside resistance genes (p=0.49). In Malawi, no patients reported macrolide use at presentation. Five of the 29 patients with aminoglycoside resistance determinants presented on a topical aminoglycoside compared with three of the 21 patients without aminoglycoside resistance determinants on their ocular surface (p=0.78). Table 2 summarizes the genes and mechanisms that confer resistance in these patient populations. Macrolide resistance is predominantly secondary to mutations in one gene group coding for 23S rRNA. Aminoglycoside resistance is due to mutations from multiple gene groups including *A16S, RRS, RRSA, RRSC* and *RRSH*. Fluoroquinolone resistance was detected in some patients in Malawi with mutations in *patA* and *patB* genes, which encode ATP-binding cassette transporters. The presence of resistance determinants for both macrolides (p=0.014) and aminoglycosides (p<0.001) was more prevalent on the ocular surface of patients in Malawi compared with patients in California.

**Table 2.** Specific mutations and mechanisms conferring AMR

Site	AMR class	Gene group	Mechanism	Accession numbers
UCSF	Aminoglycosides	A16S	Aminoglycoside-resistant 16S ribosomal subunit protein	MEG 11, MEG 2, MEG 4, MEG 5, MEG 7
	Aminoglycosides	RPSL	Aminoglycoside-resistant 16S ribosomal subunit protein	MEG 8678
	Aminoglycosides	RRS	Aminoglycoside-resistant 16S ribosomal subunit protein	MEG 8680
	Aminoglycosides	RRSC	Aminoglycoside-resistant 16S ribosomal subunit protein	MEG 6146
	Aminoglycosides	RRSH	Aminoglycoside-resistant 16S ribosomal subunit protein	MEG 6147
	MLS	MLS23S	Macrolide-resistant 23S rRNA mutation	MEG 3978, MEG 3982, MEG 3988, MEG 3992, MEG 3994, MEG 8249, MEG 8250, MEG 8251, MEG 8252, MEG 8253, MEG 8254
	Tetracyclines	TET16S	Tetracycline-resistant 16S ribosomal subunit protein	MEG 6964
Malawi	Aminoglycosides	A16S	Aminoglycoside-resistant 16S ribosomal subunit protein	MEG 11, MEG 2, MEG 3, MEG 4, MEG 5, MEG 6, MEG 7, MEG 9
	Aminoglycosides	RRS	Aminoglycoside-resistant 16S ribosomal subunit protein	MEG 8680
	Aminoglycosides	RRSA	Aminoglycoside-resistant 16S ribosomal subunit protein	MEG 6145
	Aminoglycosides	RRSC	Aminoglycoside-resistant 16S ribosomal subunit protein	MEG 6146
	Aminoglycosides	RRSH	Aminoglycoside-resistant 16S ribosomal subunit protein	MEG 6147
	Fluoroquinolones	PATA	Fluoroquinolone ABC efflux pump	MEG 5397
	Fluoroquinolones	PATB	Fluoroquinolone ABC efflux pump	MEG 5398
	MLS	MLS23S	Macrolide-resistant 23S rRNA mutation	MEG 3975, MEG 3977, MEG 3978, MEG 3988, MEG 3992, MEG 3994, MEG 8249, MEG 8250, MEG 8251, MEG 8252, MEG 8253, MEG 8254
	MLS	RLMA	23S rRNA methyltransferases	MEG 6057
	Sulfonamides	FOLP	Sulfonamide-resistant dihydropteroate synthases	MEG 2957
	Tetracyclines	TET16S	Tetracycline-resistant 16S ribosomal subunit protein	MEG 6963
	Beta-lactams	PBP2B	Penicillin binding protein	MEG 5406
	Beta-lactams	PBP2X	Penicillin binding protein	MEG 5407

AMR: antimicrobial resistance; UCSF: University of California San Francisco; MLS: macrolide-lincosamide-streptogramin B class.

It was not common for AMR gene mutations to be present in both eyes. The majority of genotypic AMR was present monocularly. At UCSF, all mutations conferring both aminoglycoside resistance and macrolide resistance were found in one eye only; no participant carried the mutations on both eyes. Aminoglycoside AMR genes were present on the conjunctiva opposite to the cornea ulcer 30% of the time. Ocular surface macrolide AMR determinants localized to the conjunctiva on the side without the corneal ulcer 13% of the time. In Malawi, 36% of patients with mutations conferring aminoglycoside resistance demonstrated the mutation on the ocular surface of both eyes. Here, 12% demonstrated aminoglycoside resistance determinants on the conjunctiva side without the corneal ulcer. In Malawi, 31% of patients with ocular surface macrolide resistance determinants carried the mutation in both eyes, with 14% of the mutations occurring only on the conjunctiva corresponding to the unaffected cornea.

To determine if the number of AMR genes on the ocular surface at presentation was associated with visual outcomes, an adjusted linear regression model compared features between patients in whom a pathogen was identified with patients without a pathogen identified (corneal ulcers that were either sterile or, presumably, completely treated prior to swabbing). In the UCSF cohort, 44 of 47 had visual acuity data recorded at their 4-wk follow-up visit. Visual acuity recorded at presentation was associated with visual acuity at 4 wk (p<0.001). In the Malawi cohort, visual acuity data at the 4-wk follow-up was available in 36 of the 50 participants. Similar to the UCSF cohort, the patients’ visual outcomes at this 4-wk follow-up were dependent on their visual acuity at presentation (p<0.001).

Additionally, comparing patients with a pathogen identified with those without a pathogen identified in the UCSF group, both the number of AMR determinants (p=0.04) and the presence of an *Acanthamoeba* corneal infection (p=0.01) predicted a worse visual acuity at 4 wk (Table 3). In Malawi, the presence of a fungal corneal infection was predictive of a worse 4-wk acuity (p=0.01), but the number of the AMR determinants was not (p=0.28). Unlike UCSF, *Acanthamoeba* was only detected in one patient in the Malawi cohort.

**Table 3.** Predictors of vision at 4 wk in presumed infectious keratitis patients with and without a pathogen identified

	Malawi	UCSF
Acuity at presentation	p<0.001	p<0.001
Number of AMR determinants	p=0.29	p=0.04
Bacteria	p=0.38	p=0.23
Fungus	p=0.01	p=0.88
Acanthamoeba	N/A	p=0.01

AMR: antimicrobial resistance; UCSF: University of California San Francisco.

N/A: not included in analysis, one acanthamoeba in this group.

## Discussion

In both San Francisco, CA, USA, and Blantyre, Malawi, gene mutations associated with AMR were commonly detected on the ocular surface of patients with infectious keratitis. Patients in Malawi are more likely to carry AMR determinants on their ocular surface than patients who presented to outpatient clinics in San Francisco in the USA. We were unable to find evidence that the patients’ current antibiotic eyedrop intake was associated with the AMR determinants detected. This appears to suggest that some selective pressure for AMR has already occurred in the communities, potentially due to prior frequent use of topical antibiotics and/or frequent use of systemic antibiotics. The patients’ medication intake could provide some possible insight into some of the selective pressure for the ocular resistance patterns observed, as it reflects the clinical practice patterns in the populations studied. This is particularly true of aminoglycosides; 12% of the patients in Malawi and 40% of patients in UCSF presented with at least one aminoglycoside eye medication. The preferred practice pattern for corneal ulcers is the initiation of broad-spectrum antibiotics. In the USA, cultures are commonly not performed for acute corneal ulcers that are non-central and <2 mm.^15^ Given that UCSF is a tertiary center, it was not surprising that most of the patients presented on eyedrops by the time they were referred for management.

The selection pressure for macrolide resistance determinants is less clear based on eyedrop-use patterns. It is possible the use of macrolides for systemic treatment of infections in the communities could be contributing to this finding.^16,17^ None of the patients in Malawi and 9% of the UCSF patient populations presented on a macrolide eyedrop. The correlation between ocular surface resistance and systemic antibiotic resistance has not been well studied. At UCSF, adult inpatient and outpatient antibiotic susceptibility data for systemic disease demonstrate macrolide phenotypic resistance rates between 25% and 42%.^18^ The phenotypic AMR rate of systemic infections to macrolides is similar to the 32% prevalence of genetic resistance determinants demonstrated in patients from the same institution on the ocular surface.

The reported phenotypic aminoglycoside resistance in systemic infections at UCSF of 9%, however, is lower than the 21% prevalence of resistance determinants on the ocular surface as determined by RNA-seq. In Malawi, a review of inpatient antimicrobial susceptibility to a variety of antibiotics demonstrated a 17–40% aminoglycoside phenotypic resistance rate, slightly lower than the 58% prevalence as determined by RNA-seq on the ocular surface.^19^ Macrolide resistance rates were not reported. Fluoroquinolone resistance rates, as studied with systemic bloodstream infections, have increased over the last decade.^20^ The reasons for increased AMR in a community are complex and numerous. Overuse of antibiotics, including overuse of extend-spectrum antibiotics, is common, often due to the unavailability of pathogen confirmation prior to prescribing, with empiric antibiotic therapy remaining a standard practice.^21–24^ Surveys in both the USA and Malawi suggest that pressures from patient expectations, along with a lack of time, to adequately explain why antibiotics are not required, contribute significantly to their overprescription.^25,26^ Easy community access to antibiotics at the community level in Malawi remains pervasive and may also be a contributing factor to the observed increased prevalence of AMR determinants detected in this population.^26–29^ Prior to the validation of trachoma's elimination as a public health problem by WHO, mass drug administration (MDA) of azithromycin had been dispensed to the adult and pediatric communities in many Malawi communities meeting the criteria for treatment.^30^ Blantyre, one of the largest cities in Malawi, and the location of this study, was not a site of MDA, but the nearby rural Mangochi district was. While it is difficult to quantify the frequency and magnitude of population spread between districts, it is also likely that people will travel to the large city for specialized eye care given a blinding infection. Also, the rate of AMR, even prior to MDA, was noted to be higher in Malawi than in other regions in Africa.^31^ The reasons for this are unclear and speculative. Additional possible contributors to increased community AMR prevalence include unregulated and overuse of antibiotics in livestock.^32,33^ Indeed, a qualitative survey of antibiotic use in the farming communities in Blantyre revealed that streptomycin (an aminoglycoside) and erythromycin (a macrolide) were the second and third most used antibiotics, respectively.^34^ Antimicrobial use in livestock is common in both the USA and Malawi with varying use and regulation within and between countries.^34,35^ Challenges in accurate reporting, variations in farming techniques and limited resources to enforce regulation contribute to the limitations of overuse assessment and correlation. Further contributors to AMR prevalence in any community may include differences in healthcare infrastructure with varying access to diagnostic tools, differences in climate and other potential environmental reservoirs of resistance, including water quality and sanitation infrastructure.

Neither the presence nor the number of AMR determinants was found to be a predictor of visual acuity for patients with fungal and bacterial corneal infections at UCSF. It is likely that visual acuity outcomes at 4 wk were not long enough to assess an ultimate vision outcome postinfection as many large central ulcers are still actively infected or inflamed at this time point. The 4-wk outcome was a standard element of data collection in the larger capriCORN study. At this 4-wk time point, specifically, if we were to quantify the impact that ocular AMR determinants could have on clinical outcomes, microbiologic resolution of corneal infection may be a better outcome to follow than visual acuity. This is because vision at 4 wk commonly cannot be best corrected at this time point in patients with severe corneal infections who may take many more weeks to months to recover. Additionally, the correlation between genotypic and phenotypic AMR on the ocular surface is confounded by the nature of direct administration of antibiotics onto the eye. This allows for a higher concentration of drug than can be achieved with systemic administration. Standard thresholds do not exist for interpreting phenotypic resistance for ocular surface drug delivery, as opposed to the thresholds determined for systemic antibiotic delivery.

The best predictor of visual acuity at 4 wk in both locations was the visual acuity at baseline. If a patient presents with poor vision, it is likely they remain visually limited throughout the course of their treatment. Similarly, it appears that the presence of acanthamoeba keratitis, commonly delayed in referral, predicted decreased visual acuity at a 4-wk follow-up visit in the USA and a fungal etiology predicted decreased visual acuity at a 4-wk follow-up visit in Malawi. This may be especially true if most fungal corneal ulcers are presumptively being treated as bacterial. Curiously, when controlling for pathogen identification, the quantity of AMR determinants was predictive of a worse visual acuity outcome in the UCSF cohort and not in the Malawi cohort. Future investigations into the host transcriptome and/or microbiome may shed more light on this finding, as it is conceivable that local environmental differences or microbial interactions could influence the relationship between AMR determinants and visual outcomes. Similarly, it is possible that variations in ocular microbiome or monocular inoculation/transmission patterns could explain the asymmetric prevalence of ocular surface AMR determinants. It is important to consider potential confounders when comparing AMR prevalence between two distinct settings that include major differences in geography and climate, variations in healthcare infrastructure, access to care and antibiotic usage patterns.

This study is limited by the comparison of only one institution in the USA and one site in sub-Saharan Africa. Additionally, phenotypic resistance was not evaluated. At the Kamuzu College of Health Sciences, culturing corneal ulcers is not a standard of care as microbiology infrastructure is lacking. In the USA, even when cultures are performed, only 50–60% of the cultures are positive, and given that >90% of the patients present to a tertiary referral clinic already on eyedrops, rates of culture positivity can be even lower.^36,37^ In the absence of a cultured organism, phenotypic resistance cannot be performed. The presence of AMR determinants, as determined by RNA-seq on the ocular surface, may not always correlate with phenotypic antimicrobial resistance, although it will most often be correlative. Many considerations, including environmental factors, levels of expression of resistant genes and the presence of other inhibitory or enhancement genes, all contribute to the ultimate phenotypic expression of AMR. Using pathogen presence as a categorical variable in our adjusted linear regression model may not accurately represent the relationship between a specific pathogen and visual acuity. However, the sample size for each pathogen at the species level was not large enough to allow for a more granular analysis. Additionally, the lack of correction for multiple comparisons could increase the risk of false positive associations; however, given the exploratory nature of this analysis, we prioritized identifying potential trends for future further confirmation and investigation. An additional limitation is that follow-up visual acuity data were only available for 4 wk as per this study's protocol and not at corneal ulcer resolution. The patient population described here was not a random sample of the population. All patients had a clinical diagnosis of infectious keratitis in one eye and presented for care. It is possible these results are not necessarily applicable to the ocular surface of a population without infectious keratitis. Additionally, patients seeking treatment at tertiary care centers often present with more advanced diseases, which could introduce selection bias and limit the generalizability of these findings to less severe cases or other settings. Finally, the sample size is small for both sites, which reduces statistical power and minimizes generalizability.

## Conclusions

Ocular surface infections are common and can be visually threatening. As determined by RNA-seq, ocular surface AMR gene mutations are common in patients with infectious keratitis. AMR genes are more prevalent on the ocular surface of patients in Malawi compared with California.

Regional surveillance for causative pathogens is important for disease preparedness. Expanding ocular surface AMR surveillance in terms of different geographic and climate coverage and over multiple time points could have important implications for clinical practice, particularly in the management of bacterial ocular infections. Identification of ocular surface AMR trends could inform empiric antibiotic decisions specific to location and potentially identify populations at risk for poorer outcomes who may warrant closer clinical follow-up. Repeat surveillance of community carriage of AMR determinants on the ocular surface in patients with keratitis may be useful in the guidance of treatment at the individual level and inform antibiotic stewardship at the community level.

## Data Availability

Data supporting the conclusions of this article will be available in the Dryad database.
